# Supplementary material for: Semi-Embedding Zn-Co3O4 Derived from Hybrid ZIFs into Wood-Derived Carbon for High-Performance Supercapacitors
Source: Molecules. 2022 Dec 5;27(23):8572. doi: 10.3390/molecules27238572 (PMC9739616; doi:10.3390/molecules27238572)
Supplement: Supplementary file 1 [file molecules-27-08572-s001.zip › molecules-2043902-supplementary.pdf]

Supporting Information

Semi-Embedding Zn-Co_3O_4 Derived from Hybrid ZIFs into Wood-derived Carbon for High-performance Supercapacitors

Wanning Xiong[†], Jie Ouyang[†], Xiaoman Wang, Ziheng Hua, Linlin Zhao Mengyao Li, Yuxin Lu, Wei Yin, Gonggang Liu, Cui Zhou, Yongfeng Luo* , Binghui Xu*

[†] The authors are equal contribution

*Corresponding authors: liugonggang@csuft.edu.cn (G.L.); yfluo@csuft.edu.cn (Y.L.)

A: Calculate the single-electrode capacitance under the three-electrode system

The volume and specific capacitance of a single electrode in a three-electrode cell configuration can be calculated according to their GCD curves by the following equations:

$$C_{(Volume)} = \frac{I \times \Delta t}{V \times \Delta U}$$

$$C_{(Specific)} = \frac{I \times \Delta t}{m \times \Delta U}$$

where I is the discharge current, Δt is the discharge time, ΔU is the potential window of the discharge process, V is the effective volume of the electrode, and m is the mass of active material.

B: Calculate the asymmetric supercapacitor capacitance under the two-electrode system

The volume and specific capacitance of asymmetric supercapacitor under the two-electrode system can be calculated according to their GCD curves by the following equations:

$$C_{(Volume)} = \frac{I \times \Delta t}{V \times \Delta U}$$

$$C_{(Specific)} = \frac{I \times \Delta t}{m \times \Delta U}$$

In this case, V is the total volume of the two electrodes, and m is the total mass of the device. The energy density (E) and power density (P) were calculated according to the following equations respectively:

$$E = \frac{1}{2} \times C \times \Delta U^2$$

$$P = \frac{E}{\Delta t}$$

where C is the volume or specific capacitance of the asymmetric supercapacitor, Δt and

ΔU are the discharge time and potential window of the discharge process.

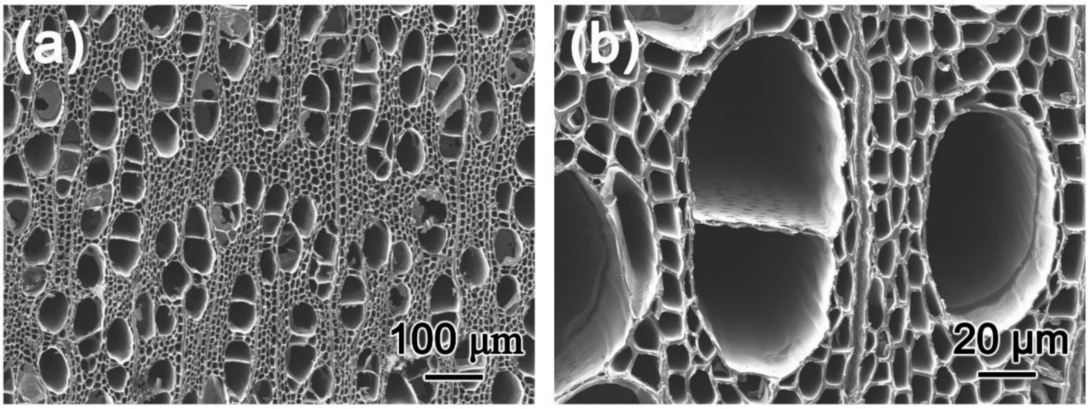

Figure S1. SEM of the poplar wood slice: perpendicular to the growth direction at different magnification times

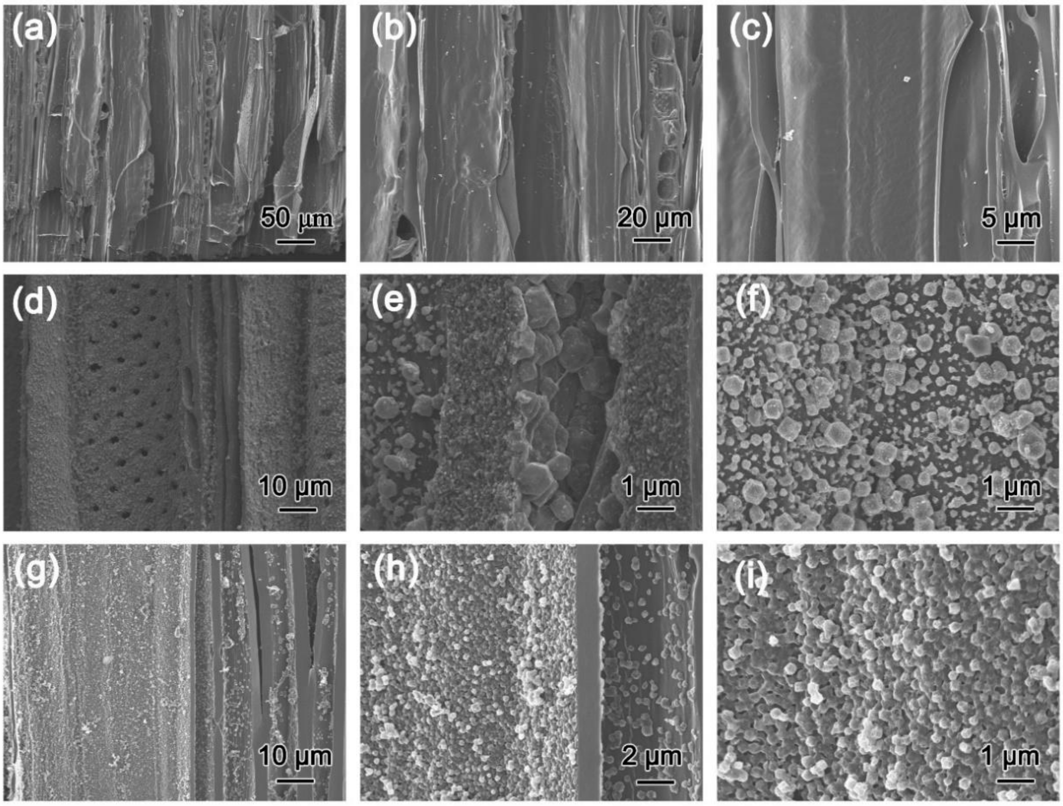

Figure S2. SEM images of nature wood (a-c), Co_3O_4@CW-230 (d-f) and Zn@CW-230 (g-i)

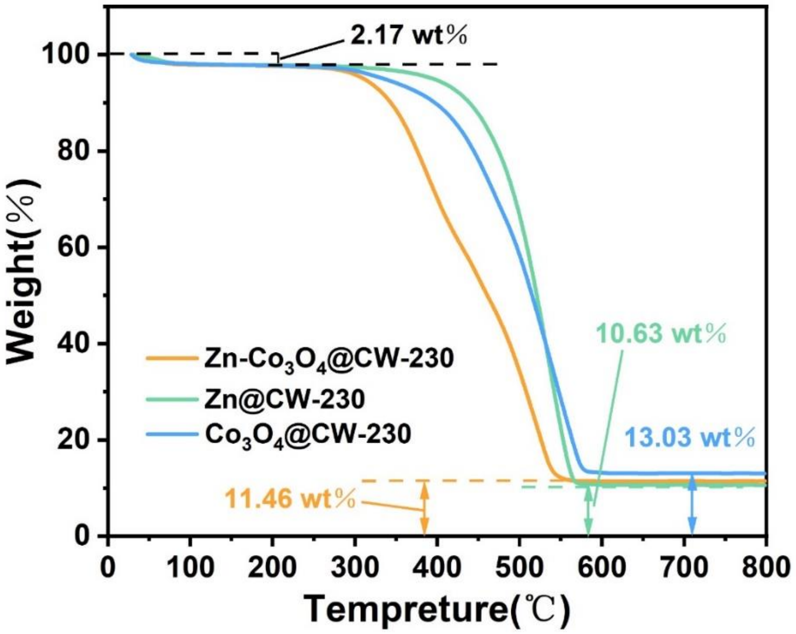

Figure S3. TGA curves of Zn@CW-230, Co_3O_4@CW-230, Zn-Co_3O_4@CW-230 composites in air.

Table S1. Analysis results of energy dispersive X-ray spectrogram

Element	Weight%	Atpmic%
C K	74.11	87.05
N K	0.76	0.77
O K	9.64	8.50
Co K	14.49	3.47
Zn K	0.98	0.21

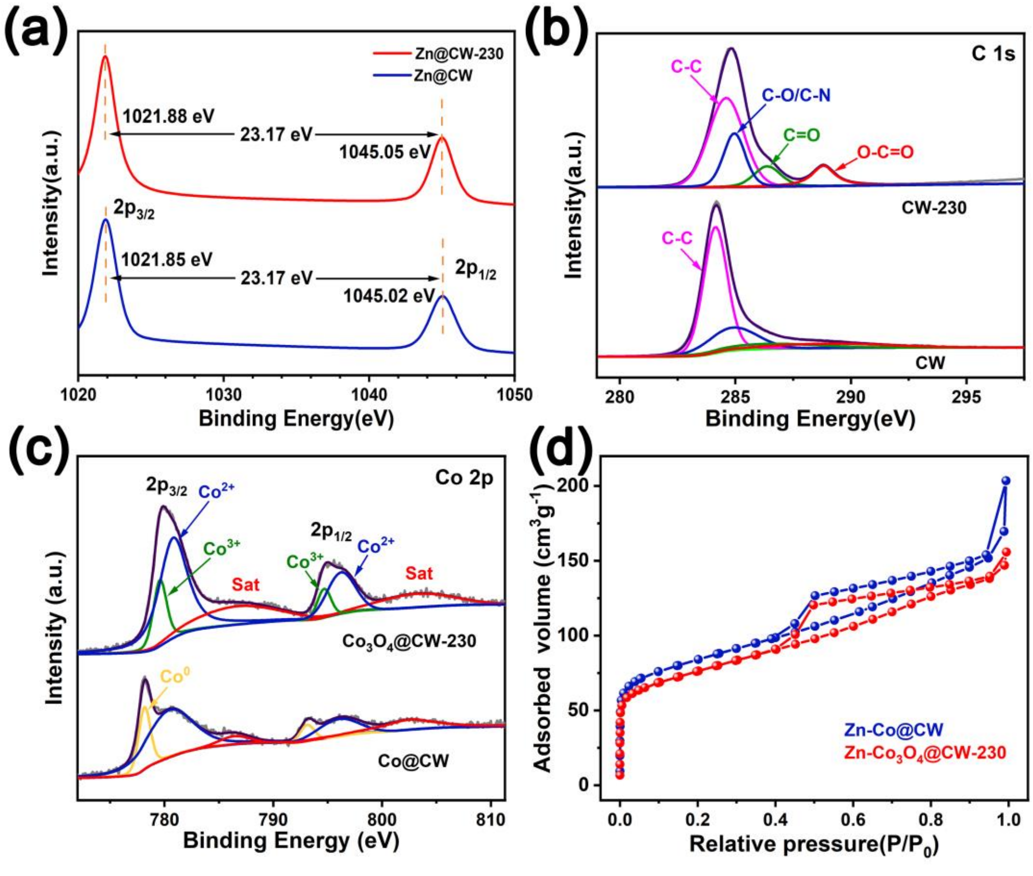

Figure S4. XPS spectra of Zn@CW and Zn@CW-230 for Zn 2p (a); CW and CW-230 for (b) C 1s; Co@CW and Co_3O_4@CW-230 for (c) Co 2p. (d) N_2 adsorption-desorption isotherm.

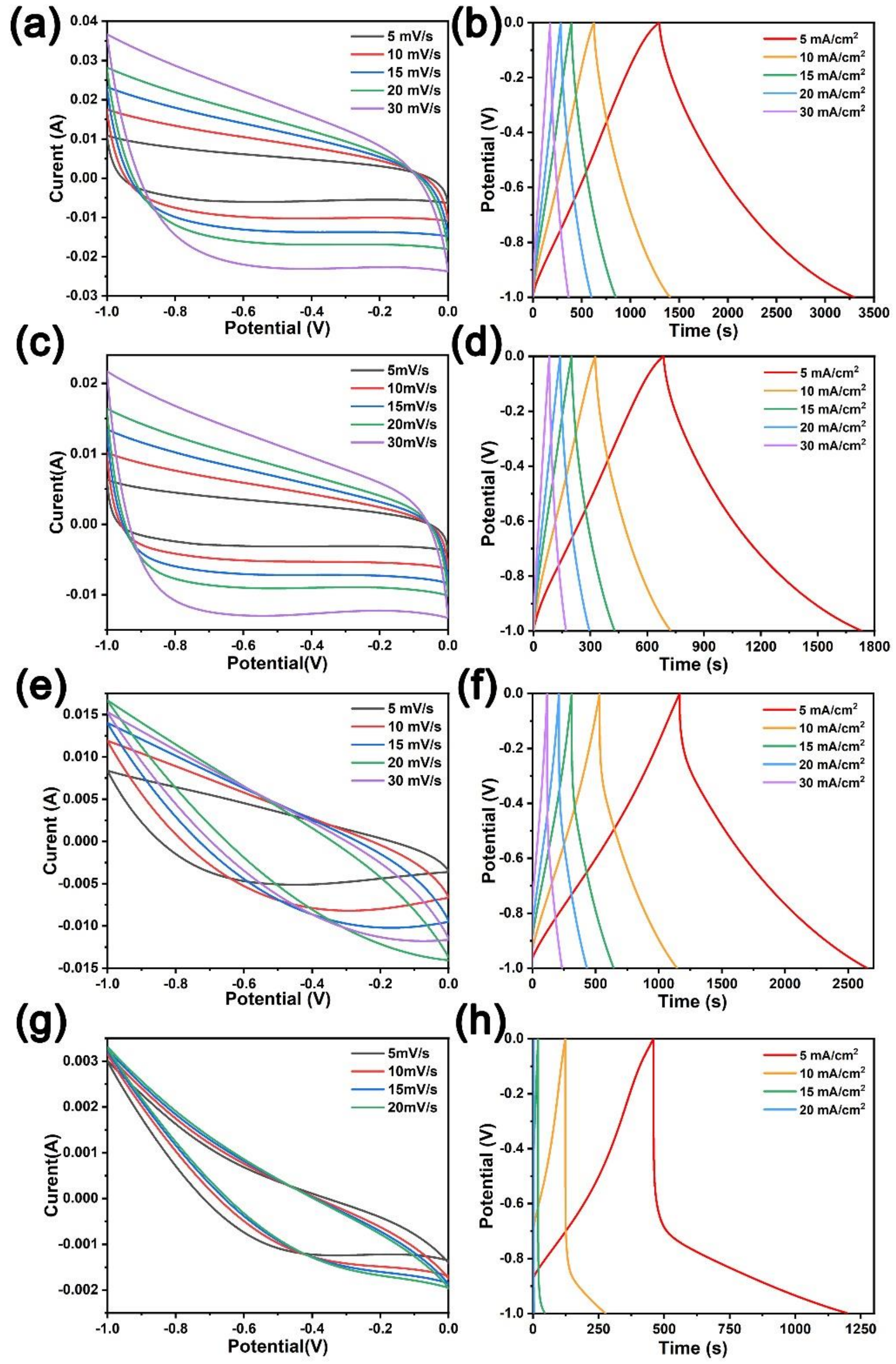

Figure S5. Cyclic voltammograms (CVs) at different scan rates and galvanostatic charge–discharge curves at different current densities of (a and b) Zn-Co@CW; (c and d) Co@CW; (e and f) Zn@CW;(g and h) CW.

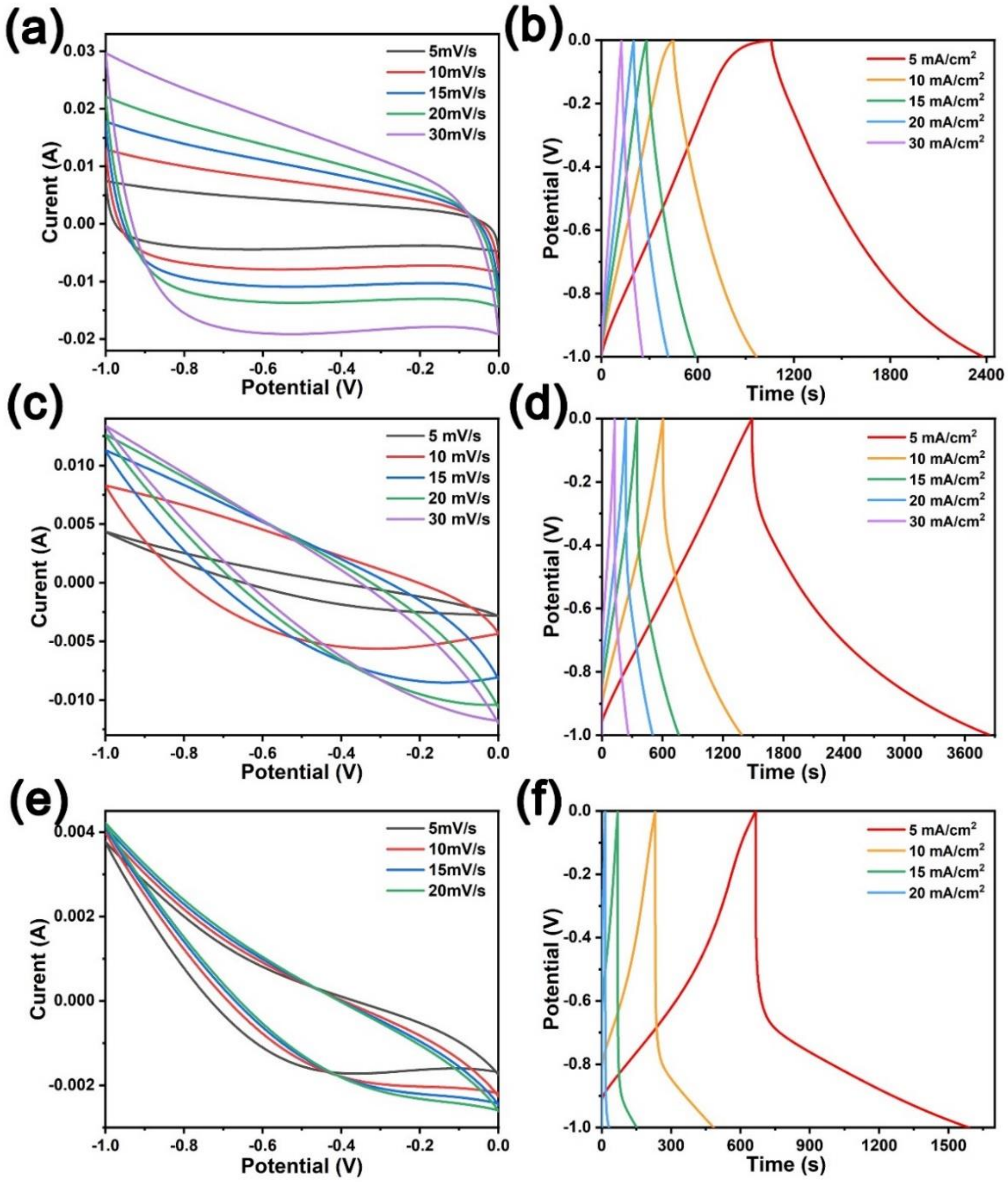

Figure S6. Cyclic voltammograms (CVs) at different scan rates and galvanostatic charge–discharge curves at different current densities of (a and b) Co_3O_4@CW-230; (c and d) Zn@CW-230; (e and f) CW-230.

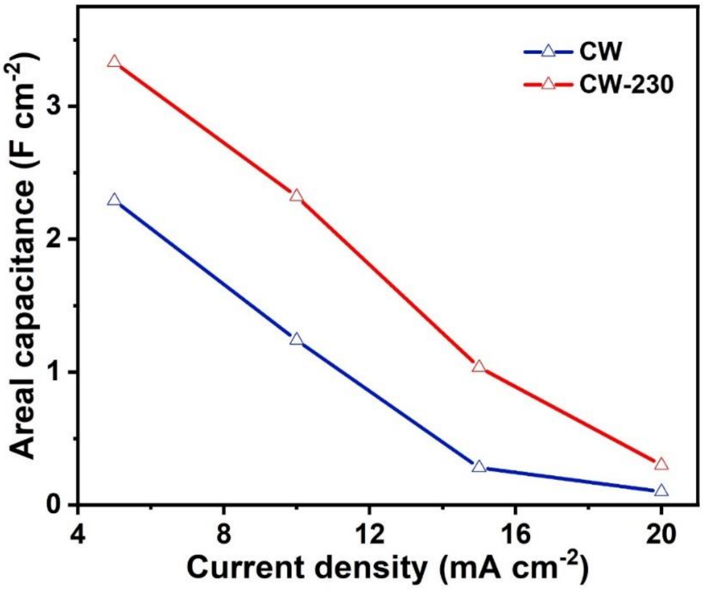

Figure S7. Specific capacitances of CW, CW-230 at different current densities.

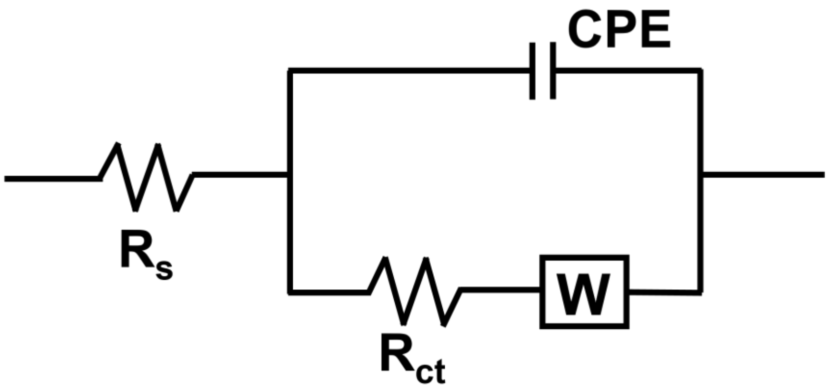

Figure S8. Equivalent circuit diagram fitted by Nyquist diagram.

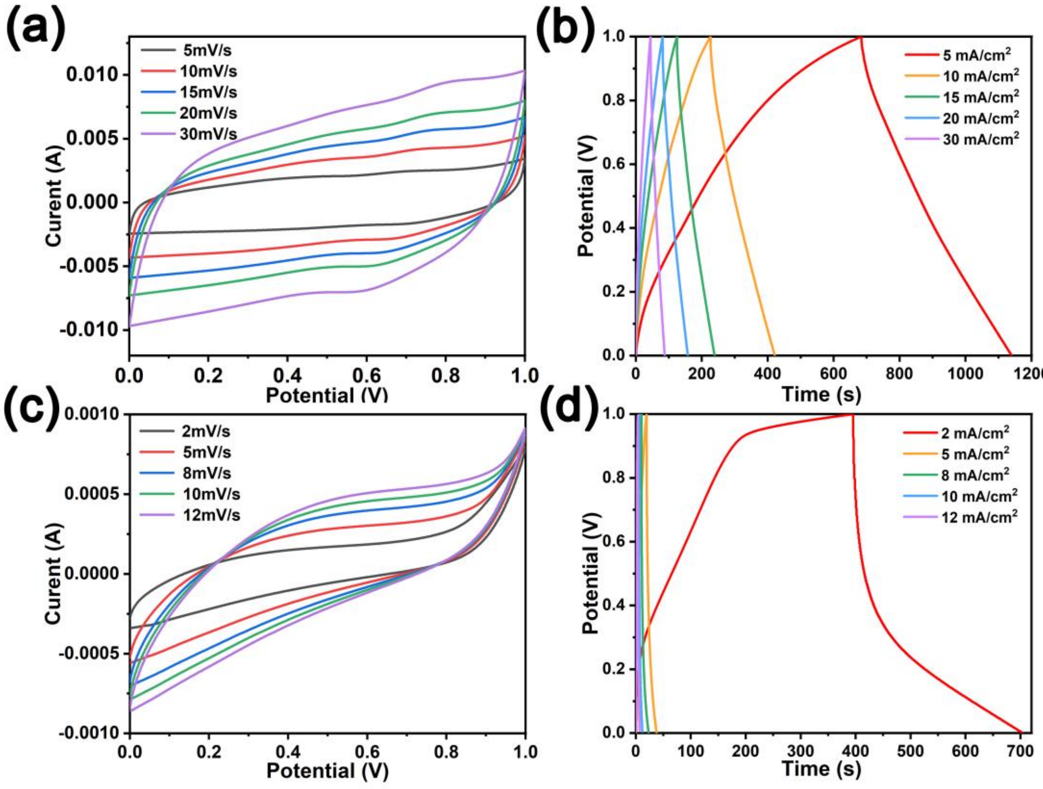

Figure S9. CVs at different scan rates and GCD curves at different current densities of (a and b) SC of Co_3O_4@CW-230. (c and d) SC of CW-230.

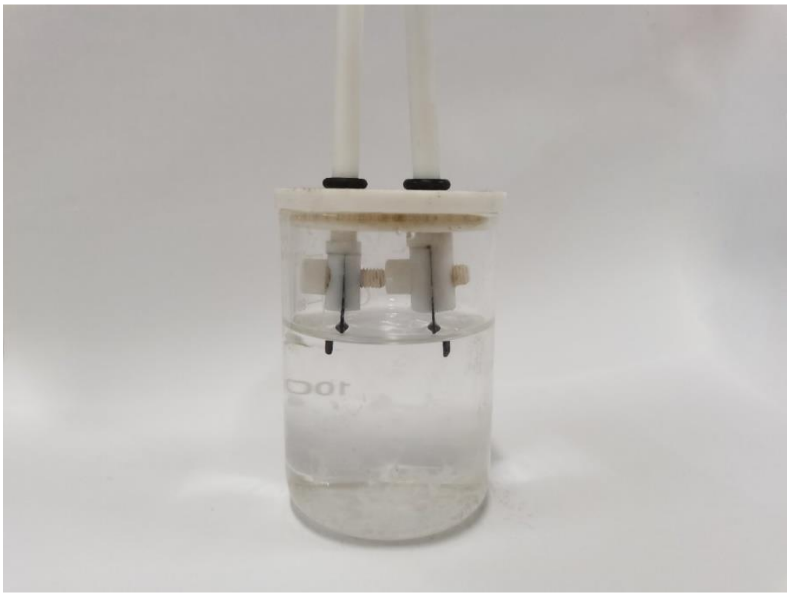

Figure S10. Photos of a two-electrode system of the Zn-Co_3O_4@CW-230 liquid-

state SC.

Table S2. Comparison of electrochemical performance of SC of Zn-Co_3O_4@CW-230 and some representative carbon-/metal oxide-based electrodes at device level.

**Electrode material**	**Volumetric Energy density ($mW\ h\ cm^{-3}$)**	**Cycles/ retention**	**Specific capacitances**	**Ref.**
CW@CoNiP-C//CW	/	5000/ 87.5%	1280 mF/cm^2, 5 mA/cm^2	[1]
NCS-ES/CW-20//AC	69.03 Wh/Kg, 862.88 W/kg	5000/ 102%	125.8 F/g, 1 A/g	[2]
MnO_2/ZnO//RGO	0.234 mW h/cm^3, 133 mW/cm^3	5000/ 98.4%	250 mF/cm^2, 10 mV/s	[3]
CoP/MnO_2/CC	0.69 mW h/cm^3, 114.2 mW/cm^3	5000/ 82%	571.3 mF/cm^2, 1 mA/cm^2	[4]
Fe_2O_3/MnO_2	0.55 mW h/cm^3, 139.1 mW/cm^3	5000/ 84%	180.4 mF/cm^2, 1 mA/cm^2	[5]
Co_3O_4/graphene hybrid fibers	/	10000/ 72.7%	196.3 mF/cm^2, 0.2 mA/g	[6]
$CoSe_2$//CC	0.588 mW h/cm^3, 282 mW/cm^3	2000/ 94.8%	332 mF/cm^2, 1 mA/cm^2	[7]
C-ZIF-8@ACW	4.4 mW h/cm^3 4.0mW/cm^3	10000/ 96.8%	3935 mF/cm^2, 1 mA/cm^2	[8]
Zn-Co_3O_4@CW-230	6.0 mW h $/cm^3$ 41.7 mW/cm^3	10000/ 97.3%	2610 mF/cm^2, 5 mA/cm^2	***This work***

Reference

1. Tian, R.; Duan, C.; Feng, Y.; Cao, M.; Yao, J., Metal Organic Framework-Based CoNi Composites on Carbonized Wood as Advanced Freestanding Electrodes for Supercapacitors. *Energy & Fuels* **2021,** 35, (5), 4604-4608.
2. Rafiq, M. I.; Farid, T.; Zhou, J.; Ali, A.; Tang, J.; Tang, W., Carbonized wood-supported hollow NiCo2S4 eccentric spheres for high-performance hybrid supercapacitors. *Journal of Alloys and Compounds* **2019,** 811, 151858.
3. Zilong, W.; Zhu, Z.; Qiu, J.; Yang, S., High performance flexible solid-state asymmetric supercapacitors from MnO2/ZnO core–shell nanorods//specially reduced graphene oxide. *J. Mater. Chem. C* **2014,** 2, (7), 1331-1336.
4. Zheng, Z.; Retana, M.; Hu, X.; Luna, R.; Ikuhara, Y. H.; Zhou, W., Three-Dimensional Cobalt Phosphide Nanowire Arrays as Negative Electrode Material for Flexible Solid-State Asymmetric Supercapacitors. *ACS Appl Mater Interfaces* **2017,** 9, (20), 16986-16994.
5. Yang, P.; Ding, Y.; Lin, Z.; Chen, Z.; Li, Y.; Qiang, P.; Ebrahimi, M.; Mai, W.; Wong, C. P.; Wang, Z. L., Low-cost high-performance solid-state asymmetric supercapacitors based on MnO2 nanowires and Fe2O3 nanotubes. *Nano Lett* **2014,** 14, (2), 731-736.
6. Wang, W.; Yuan, Y.; Yang, J.; Meng, L.; Tang, H.; Zeng, Y.; Ye, Z.; Lu, J., Hierarchical core–shell Co3O4/graphene hybrid fibers: potential electrodes for supercapacitors. *J Mater Sci* **2018,** 53, (8), 6116-6123.
7. Yu, N.; Zhu, M.-Q.; Chen, D., Flexible all-solid-state asymmetric supercapacitors with three-dimensional CoSe2/carbon cloth electrodes. *Journal of Materials Chemistry A* **2015,** 3, (15), 7910-7918.
8. Zhang, W.; Li, M.; Zhong, L.; Huang, J.; Liu, M., A family of MOFs@Wood-Derived hierarchical porous composites as freestanding thick electrodes of solid supercapacitors with enhanced areal capacitances and energy densities. *Mater Today Energy* **2022,** 24.
